# Suppression of Hsp90 expression in *Aspergillus fumigatus* enhances sensitivity to oxidative stress and activates host cell NF-κB p65 and ERK signaling pathways

**DOI:** 10.1128/mbio.00094-26

**Published:** 2026-03-16

**Authors:** Lingyun Song, Deqi Jiang, Xiaogang Zhou, Xiaokang Yi, Shihan Jia, Yasheng Li, Lei Zhang, Jiabin Li, Jinxing Song

**Affiliations:** 1 The Key Laboratory of Biotechnology for Medicinal and Edible Plant Resources of Jiangsu Province, School of Life Sciences, Jiangsu Normal University 216827, Xuzhou, China; 2 Department of Infectious Diseases & Anhui Province Key Laboratory of Infectious Diseases, The First Affiliated Hospital of Anhui Medical University 36639, Hefei, China; 3 Basic Medical Research Center, Shandong Provincial Third Hospital 582805, Jinan, China; 4 Anhui Key Laboratory of Infection and Immunity, Department of Microbiology, School of Basic Medicine, Bengbu Medical University 74539 , Bengbu, Anhui, People's Republic of China; Medizinische Universitat Graz, Graz, Austria

**Keywords:** Hsp90, *Aspergillus fumigatus*, oxidative stress, immune responses, fungal pathogens

## Abstract

This study investigates the pivotal role of the molecular chaperone Hsp90 in the growth, development, and immune modulation of the opportunistic pathogen *Aspergillus fumigatus*. Using a Tet-on system-based conditional expression strain, *Tet-hsp90*, we demonstrate that Hsp90 is essential for the growth and viability of *A. fumigatus*. In strains with suppressed Hsp90 expression, we observe a significant reduction in antioxidant capacity, an increase in intracellular reactive oxygen species levels, and a marked decrease in the activity of key antioxidant enzymes, such as superoxide dismutase and catalase, highlighting Hsp90’s critical role in regulating oxidative stress tolerance. Furthermore, the absence of Hsp90 leads to disruptions in metal ion metabolism, including copper, iron, and manganese, which further compromises the fungal cell’s ability to manage oxidative stress. Immune response analysis revealed that after Hsp90 inhibition, spores significantly increased the expression of pro-inflammatory cytokines, such as interleukin-1β (IL-1β), IL-6, and tumor necrosis factor alpha, in host cells. Further research showed that, upon Hsp90 inhibition, spores enhanced the phosphorylation of NF-κB p65 and ERK proteins, thereby activating these key signaling pathways to upregulate immune responses. In conclusion, this study underscores the multifaceted regulatory roles of Hsp90 in oxidative stress resistance, metal ion homeostasis, and immune system activation, providing valuable insights for the development of novel antifungal therapeutic strategies targeting Hsp90.

**IMPORTANCE:**

The significance of this study lies in its focused and in-depth analysis of the critical roles of the molecular chaperone Hsp90 in oxidative stress tolerance, metal ion homeostasis, and immune response regulation in *Aspergillus fumigatus*. Utilizing the Tet-on system to precisely modulate Hsp90 expression, this research demonstrates that Hsp90 is essential for the growth, development, and pathogenicity of this major opportunistic pathogen. Specifically, the study highlights Hsp90's crucial role in sustaining antioxidant enzyme activity, maintaining metal ion balance, and regulating immune responses—key factors that enable the fungus to survive and infect the host. These findings provide novel insights into the molecular mechanisms that underlie *A. fumigatus*'s resistance to oxidative stress and its evasion of host immunity, positioning Hsp90 as a promising therapeutic target for treating fungal infections. Furthermore, the results open new avenues for future investigations into the role of Hsp90 in fungal pathogenesis and immune modulation, potentially guiding the development of targeted antifungal therapies.

## OBSERVATION

*Aspergillus fumigatus* is an important opportunistic pathogen, and its interaction with the host immune system is complex and multifaceted. Host immune cells primarily kill *A. fumigatus* conidia through reactive oxygen species (ROS) generated by the NADPH oxidase system (1–3). The mechanism involves oxidative damage to the conidial cell wall, DNA, and proteins, ultimately leading to conidial death (4). In response to the oxidative stress within host cells, *A. fumigatus* has developed various mechanisms to enhance its antioxidant capacity, partially evading immune cell attacks (1, 5, 6). Current research indicates that the resistance of *A. fumigatus* to oxidative stress is closely linked to its pathogenicity (1, 7). Hsp90 is a highly conserved molecular chaperone that facilitates the proper folding and function of hundreds of client proteins, many of which play crucial roles in signal transduction networks. Studies have shown that Hsp90 plays a key role in the pathogenesis of opportunistic fungal pathogens, particularly in regulating morphogenesis, virulence, and temperature adaptation in *Candida albicans* (8–11). However, the specific mechanisms by which Hsp90 participates in oxidative stress response and immune regulation in pathogenic fungi remain not fully understood.

**Fig 1:**
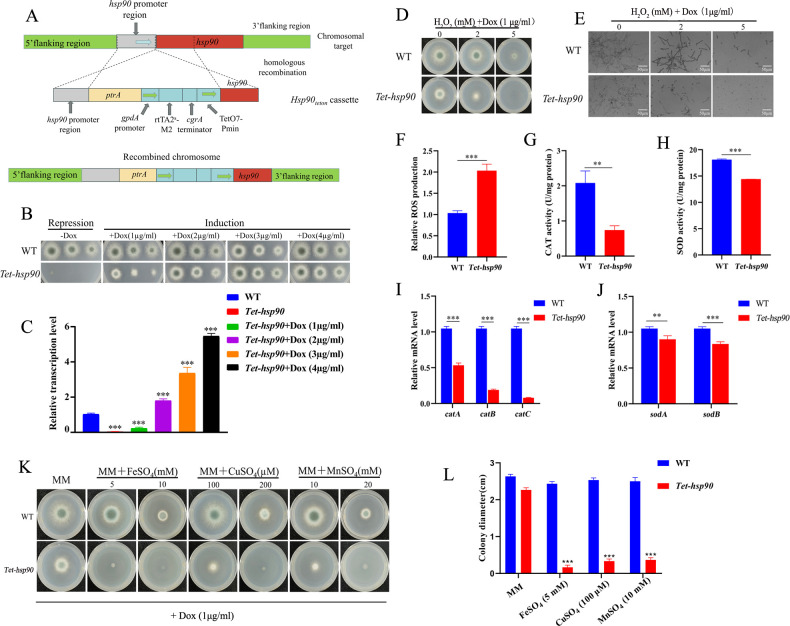
Hsp90 is essential for growth, oxidative stress tolerance, and metal ion homeostasis in *Aspergillus fumigatus*. (**A**) The endogenous promoter of the *hsp90* gene was replaced by a doxycycline-inducible promoter system through homologous recombination. (**B**) Colony morphology of WT and *Tet-hsp90* strains cultured on MM with or without doxycycline (Dox) supplementation (0–4 µg/mL). Strains were incubated at 37°C for 48 h before imaging. Suppression of Hsp90 under non-inductive conditions (−Dox) resulted in lethal growth defects, while growth was restored in a dose-dependent manner under inductive conditions (+Dox). (**C**) Quantitative RT-PCR analysis of *hsp90* transcription levels in WT and *Tet-hsp90* strains under different Dox concentrations. Transcription increased with rising Dox concentrations and surpassed WT baseline levels at 3–4 µg/mL. ***P < 0.001. (**D**) Growth phenotypes of WT and *Tet-hsp90* strains exposed to increasing concentrations of H_2_O_2_ (0, 2, and 5 mM). The medium contained 1 μg/mL Dox to induce low-level expression of *hsp90*. Strains were incubated at 37°C for 48 h before imaging. Under oxidative stress, the *Tet-hsp90* strain showed a reduced colony size compared with WT. (**E**) Growth phenotypes of WT and *Tet-hsp90* strains exposed to increasing concentrations of H_2_O_2_ (0, 2, and 5 mM) in the presence of 1 μg/mL Dox. The strains were observed after 10 h of incubation in liquid culture. At 0 mM H_2_O_2_, both WT and *Tet-hsp90* strains exhibited similar growth patterns, with WT forming a dense network of hyphae. Under oxidative stress (2 and 5 mM H_2_O_2_), the *Tet-hsp90* strain showed significantly reduced growth, while the WT strain continued to grow robustly. The scale bar represents 50 µm. (**F**) Intracellular ROS levels measured in WT and *Tet-hsp90* strains following oxidative stress. Hsp90 suppression led to markedly higher ROS accumulation. ***P < 0.001. (**G and H**) Antioxidant enzyme activities in WT and *Tet-hsp90* strains. Both CAT and SOD activities were significantly reduced in the *Tet-hsp90* strain compared with WT. **P < 0.01 and ***P < 0.001. (**I and J**) Expression levels of oxidative stress-related genes (*catA*, *catB*, and *catC*) and superoxide dismutase genes (*sodA* and *sodB*) in WT and *Tet-hsp90* strains analyzed by qRT-PCR. Hsp90 suppression resulted in strong downregulation, especially of *catA* and *catB*. ACT1 was used as the internal control. All experiments were independently repeated at least three times. Data are presented as mean ± SD. Statistical significance was determined by Student’s *t*-test, with significance indicated as **P < 0.01 and ***P < 0.001. (**K**) Growth of WT and *Tet-hsp90* strains in MM medium supplemented with increasing concentrations of FeSO_4_, CuSO_4_, and MnSO_4_, along with 1 μg/mL Dox to induce low-level expression of *hsp90*. Strains were incubated at 37°C for 48 h before imaging. The *Tet-hsp90* strain exhibited severe growth inhibition under excess metal ion conditions, indicating impaired metal ion homeostasis. (**L**) Colony diameter of WT and *Tet-hsp90* strains grown in MM medium supplemented with FeSO_4_ (5 mM), CuSO_4_ (100 μM), and MnSO_4_ (10 mM). The medium also contained 1 μg/mL Dox to induce low-level expression of *hsp90* in the *Tet-hsp90* strain. The colony diameter was measured in centimeters. Data are presented as mean ± standard deviation (SD). Statistical significance was determined by Student’s *t*-test, with significance indicated as ***P < 0.001. The colony growth images of the *Tet-hsp90* strain on MM+ (1 μg/mL Dox) shown in Fig. 1D and K were derived from the same representative image, as both panels represent the same experimental condition. Therefore, the same representative image was used for illustrative purposes in Fig. 1.

To elucidate the central role of the molecular chaperone Hsp90 in the growth and development of *A. fumigatus*, we constructed a conditional expression strain, *Tet-hsp90*, based on the Tet-on system (Fig. 1A). The construction strategy and experimental procedures are described in detail in the Materials and Methods section of the Supplemental material. The construction of the *Tet-hsp90* conditional expression strain allowed for the precise regulation of Hsp90 expression, confirming its indispensable role in the growth and development of *A. fumigatus* (Fig. 1B). Under non-inductive conditions (−Dox), the suppression of Hsp90 expression led to lethal growth defects, suggesting that Hsp90 may be crucial for processes such as cell cycle regulation, signal transduction, and morphogenesis. This is consistent with the known function of Hsp90 in stabilizing client proteins involved in cellular processes. Under inducible conditions (+Dox), doxycycline restored normal colony formation, confirming that the observed growth defects were directly related to Hsp90 suppression (Fig. 1B and C). The dose-dependent restoration of growth further emphasizes the fine-tuning ability of the Tet-on system, providing a robust model for further studies of Hsp90 function in *A. fumigatus*.

A key finding of this study is the important role of Hsp90 in oxidative stress response. When exposed to gradually increasing concentrations of H_2_O_2_, the *Tet-hsp90* strain exhibited significantly smaller colonies, indicating the critical role of Hsp90 in mitigating oxidative damage (Fig. 1D and E). At a concentration of 5 mM H_2_O_2_, the *Tet-hsp90* strain almost ceased growth, while the wild-type (WT) strain continued to form larger colonies, highlighting the robust oxidative stress tolerance of wild-type *A. fumigatus*. This observation is consistent with the broader concept that high-virulence pathogens, including *A. fumigatus*, possess stronger antioxidant capabilities, enabling them to better counteract the host immune response. The defect in oxidative stress resistance due to Hsp90 suppression suggests that Hsp90 may play a role by directly stabilizing antioxidant enzymes or regulating the expression of antioxidant-related genes. To further elucidate how Hsp90 influences oxidative stress tolerance, we examined intracellular ROS levels and analyzed antioxidant enzyme activity. The ROS levels were measured using 2′,7′-dichlorodihydrofluorescein diacetate (H2DCFDA), and the detailed procedure is described in the Materials and Methods section of the Supplemental material. Compared to the wild-type strain, the *Tet-hsp90* strain exhibited significantly higher levels of ROS (Fig. 1F), indicating that Hsp90 suppression impairs the cell’s ability to clear ROS. This could be attributed to the failure in stabilizing antioxidant enzymes, such as superoxide dismutase (SOD) and catalase (CAT), both of which are crucial for detoxifying ROS. The SOD and CAT activities in the *Tet-hsp90* strain were significantly lower than those in the wild-type strain (Fig. 1G and H), supporting the hypothesis that Hsp90 plays a role in maintaining antioxidant enzyme function. Additionally, the expression of antioxidant-related genes (such as *catA*, *catB*, *catC*, *sodA*, and *sodB*) was reduced in the *Tet-hsp90* strain (Fig. 1I and J), further reinforcing the idea that Hsp90 regulates the expression of oxidative stress genes.

The normal function of metal-dependent enzymes such as SOD and CAT is critical for antioxidant defense. As these enzymes require metal ions like copper (Cu²^+^), iron (Fe²^+^), and manganese (Mn²^+^) as cofactors to exert their catalytic activity (12–14), disruptions in the metabolism of these metal ions can severely impair the activity of SOD and CAT enzymes, thereby exacerbating oxidative stress and increasing cellular damage. Moreover, studies also have shown that an imbalance in these metal ions, particularly their excessive accumulation, can intensify oxidative stress, leading to cellular damage. For example, the overaccumulation of iron can trigger the Fenton reaction, generating highly reactive free radicals that cause oxidative damage to DNA, proteins, and lipids (15, 16); copper metabolism abnormalities can disrupt the intracellular redox balance, further increasing oxidative stress through free radical generation (17, 18); and both manganese deficiency and excess can weaken the antioxidant system, increasing the cell’s sensitivity to oxidative stress (19, 20). Therefore, the balance of these metal ions is crucial for maintaining cellular antioxidant defense. Based on this, we hypothesize that Hsp90 may play a role in regulating the balance of copper, iron, and manganese ion metabolism, thus contributing to the cell’s oxidative stress defense. Our results from the metal stress experiment support this hypothesis. In the experiment, by adding different concentrations of FeSO_4_, CuSO_4_, and MnSO_4_ to the culture medium, we observed that the *Tet-hsp90* strain exhibited significantly impaired growth under metal stress, especially at higher concentrations of metal ions (Fig. 1K and L). These results suggest that Hsp90 likely responds to oxidative stress by correctly regulating the homeostasis of copper, iron, and manganese ions.

In conclusion, this study significantly advances our understanding of the core role of Hsp90 in oxidative stress tolerance, copper, iron, and manganese ion homeostasis, and pathogenicity in *A. fumigatus*. The results demonstrate that Hsp90 is indispensable for regulating antioxidant enzyme activity, the expression of oxidative stress-related genes, and maintaining the homeostasis of copper, iron, and manganese ions. These factors collectively enhance the survival ability of *A. fumigatus* in the host’s high oxidative stress environment. These findings open the door for further studies into the molecular mechanisms of Hsp90 in oxidative stress response and its potential as a therapeutic target for fungal infections.

**Fig 2:**
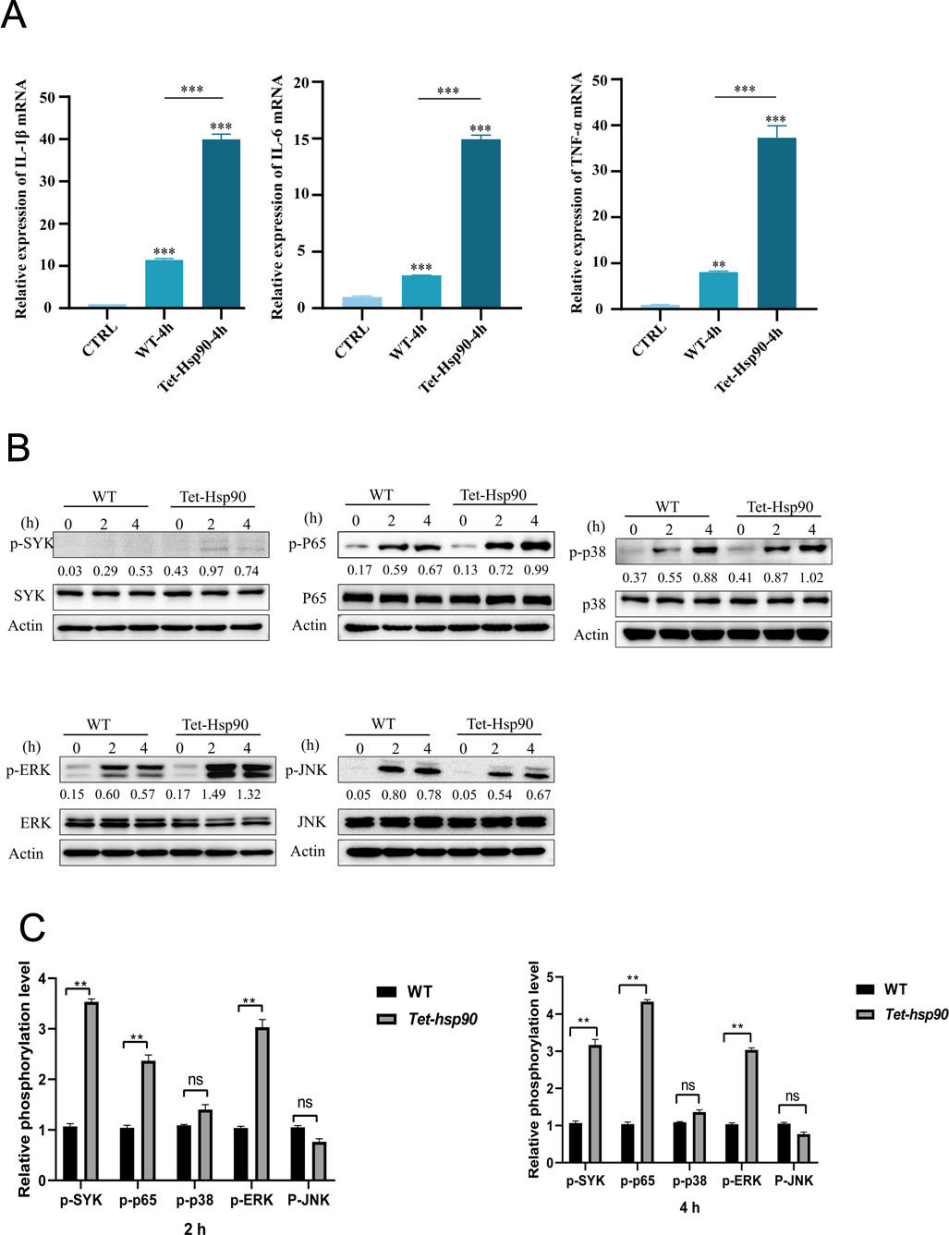
Suppression of Hsp90 expression in *Aspergillus fumigatus* enhances pro-inflammatory cytokine production and activates host NF-κB p65 and ERK signaling pathways. (**A**) Relative mRNA expression of pro-inflammatory cytokines in host cells 4 h after infection with WT or *Tet-hsp90* spores compared to uninfected CTRL. Levels of IL-1β, IL-6, and TNF-α were quantified by qRT-PCR and normalized to β-actin. Hsp90 suppression (Tet-Hsp90-4 h) resulted in a marked up-regulation of all three cytokines relative to CTRL and WT. Data are presented as mean ± SD (*n* = 3). Statistical significance was assessed by Student’s *t*-test; **P < 0.01 and ***P < 0.001. (**B**) Western blot analysis of phosphorylation levels of key immune signaling proteins in host cells infected with WT or Tet-Hsp90 spores for 0, 2, or 4 h. Phosphorylated proteins (p-SYK, p-P65, p-p38, p-ERK, and p-JNK) and total forms of SYK, P65, p38, ERK, and JNK were detected using specific antibodies; β-actin served as the loading control. Numbers below each blot indicate densitometric ratios of phosphorylated to total proteins, normalized to β-actin. Hsp90 suppression significantly enhanced phosphorylation of NF-κB p65 and ERK at both 2 h and 4 h, while phosphorylation of SYK, p38, and JNK showed no substantial change. All experiments were independently repeated at least three times with similar results. Data shown are representative blots and corresponding quantitative analyses. (**C**) Quantification of the relative phosphorylation levels of the indicated signaling molecules at 2 and 4 h post-infection, normalized to the corresponding total protein levels and expressed relative to the WT group. Data are presented as mean ± SD from at least three independent experiments. Statistical significance was determined using appropriate statistical tests. ns, not significant; **P < 0.01.

Finally, we conducted an in-depth analysis of the regulatory role of *Tet-hsp90* conidia in the host immune response, focusing on their ability to influence host immunity by modulating the expression of inflammatory cytokines. Quantitative real-time polymerase chain reaction (qRT-PCR) analysis was performed to quantify the mRNA levels of the pro-inflammatory cytokines interleukin-1β (IL-1β), IL-6, and tumor necrosis factor alpha (TNF-α) in macrophages 4 h after infection with control (CTRL), wild-type (WT), or *Tet-hsp90* conidia. The results showed that, compared with the CTRL group, infection with WT conidia significantly upregulated the transcription of all three pro-inflammatory cytokines. In contrast, infection with *Tet-hsp90* conidia resulted in a further and more pronounced increase in the expression of these cytokines (Fig. 2A), indicating that *Tet-hsp90* conidia possess a stronger capacity to induce host pro-inflammatory responses. In summary, these findings demonstrate that suppression of Hsp90 expression in conidia markedly enhances the transcriptional response of pro-inflammatory cytokines in host cells. This enhanced inflammatory response may be associated with alterations in the cell wall structure of *Tet-hsp90* conidia, thereby facilitating more effective immune recognition and activation of host inflammatory responses.

To further elucidate the molecular mechanisms by which *Tet-hsp90* conidia enhance host immune responses, we performed Western blot analysis to examine the phosphorylation levels of key molecules involved in several classical immune signaling pathways. Macrophages were infected with WT or *Tet-hsp90* conidia for 2 and 4 h, and changes in the phosphorylation status of relevant signaling proteins were subsequently analyzed. Given that SYK (spleen tyrosine kinase) plays a critical role in immune cell activation (21), we first investigated the activation of the SYK signaling pathway. The results showed that, compared with the WT group, *Tet-hsp90* infection induced significant changes in SYK phosphorylation at both 2 and 4 h post-infection (Fig. 2B and C). However, the overall phosphorylation level of SYK remained relatively low. These results suggest that SYK may participate in the immune-enhancing effects mediated by *Tet-hsp90* conidia, but it is unlikely to be the predominant signaling pathway involved. As NF-κB p65 is a key transcription factor in immune and inflammatory responses (22), we next examined the phosphorylation status of NF-κB p65. The results demonstrated that NF-κB p65 phosphorylation was markedly enhanced in the *Tet-hsp90* infection group. At both 2 and 4 h post-infection, the p-p65 levels in the *Tet-hsp90* group were significantly higher than those in the WT group (Fig. 2B and C), indicating that *Tet-hsp90* conidia effectively promote activation of the NF-κB signaling pathway. This finding is highly consistent with the significantly increased transcription levels of pro-inflammatory cytokines shown in Fig. 2A, suggesting that NF-κB signaling plays a central role in *Tet-hsp90*-induced immune enhancement. Considering the important role of the MAPK signaling pathway in immune regulation, we further analyzed the phosphorylation levels of p38, JNK, and ERK. The results showed that although phosphorylation of p38 and JNK increased over time following infection, no significant differences were observed between the WT and *Tet-hsp90* groups (Fig. 2B and C), indicating that these pathways are unlikely to be the major mediators of *Tet-hsp90*-induced immune regulation. In contrast, ERK phosphorylation was significantly increased at 4 h post-infection in the *Tet-hsp90* group and was markedly higher than that in the WT group (Fig. 2B and C), suggesting that Tet-hsp90 conidia can robustly activate the ERK signaling pathway during the later stages of infection. Taken together, these results indicate that *Tet-hsp90* conidia enhance host immune and inflammatory responses primarily through activation of the NF-κB and ERK signaling pathways, rather than through SYK, p38, or JNK pathways. Given the important roles of the ERK pathway in cell growth, differentiation, and stress responses (23), its activation during immune responses may contribute to enhanced immune cell activation and regulation of inflammation. In conclusion, *Tet-hsp90* conidia regulate host immune and inflammatory responses mainly by activating the NF-κB p65 and ERK signaling pathways, thereby promoting the expression of pro-inflammatory cytokines. These findings provide important experimental evidence for further investigation into the role of Hsp90 in host immune regulation and offer a novel perspective for exploring Hsp90 as a potential therapeutic target in antifungal strategies.

Based on the findings of this study, Hsp90 emerges as a promising therapeutic target for antifungal intervention against *A. fumigatus*. Our results demonstrate that Hsp90 is indispensable for fungal growth, oxidative stress tolerance, metal ion homeostasis, and immune evasion. Suppression of Hsp90 not only severely compromises fungal viability and antioxidant defense but also enhances host immune recognition and pro-inflammatory responses through activation of NF-κB and ERK signaling pathways. These combined effects suggest that targeting Hsp90 could simultaneously weaken fungal stress resistance and potentiate host immune-mediated clearance.

Importantly, Hsp90 inhibition resulted in increased intracellular ROS accumulation, reduced antioxidant enzyme activity, and disrupted copper, iron, and manganese homeostasis, all of which are critical determinants of fungal survival within the oxidative environment of host immune cells. Such multifaceted vulnerability highlights the advantage of Hsp90 as a central regulatory node, where pharmacological inhibition may exert broad antifungal effects rather than targeting a single downstream pathway. Moreover, the enhanced induction of pro-inflammatory cytokines observed upon *Tet-hsp90* conidial infection suggests that Hsp90 suppression may improve host immune responsiveness, potentially synergizing with innate immune mechanisms to eliminate fungal pathogens more efficiently.

Previous studies have shown that Hsp90 inhibitors can enhance the efficacy of existing antifungal drugs and reduce fungal virulence in other pathogenic fungi (24, 25). Our findings extend this concept to *A. fumigatus*, providing mechanistic evidence that Hsp90 inhibition disrupts both fungal stress adaptation and immune modulation. Therefore, Hsp90 represents an attractive candidate for combination antifungal therapies, in which Hsp90 inhibitors could sensitize fungi to oxidative damage while amplifying host immune defenses. In summary, this study not only elucidates the central role of Hsp90 in oxidative stress response and immune regulation in *A. fumigatus*, but also provides a strong theoretical and experimental basis for exploring Hsp90 as a novel therapeutic target in antifungal treatment strategies.

## SUPPLEMENTAL MATERIAL

Supplemental material mbio.00094-26-s0001.docx Additional experimental details.

ASM does not own the copyrights to Supplemental Material that may be linked to, or accessed through, an article. The authors have granted ASM a non-exclusive, world-wide license to publish the Supplemental Material files. Please contact the corresponding author directly for reuse.
