# Supplementary material for: Suppression of Hsp90 expression in Aspergillus fumigatus enhances sensitivity to oxidative stress and activates host cell NF-κB p65 and ERK signaling pathways
Source: mBio. 2026 Mar 16;17(4):e00094-26. doi: 10.1128/mbio.00094-26 (PMC13059757; doi:10.1128/mbio.00094-26)
Supplement: Supplemental material — Additional experimental details. [file mbio.00094-26-s0001.docx]

Supplemental Material

**Suppression of Hsp90 Expression in *Aspergillus fumigatus* Enhances Sensitivity to Oxidative Stress and Activates Host Cell NF-κB p65 and ERK Signaling Pathways**

Lingyun Song^1,2#^ Deqi Jiang^3#^ Xiaogang Zhou^4#^ Xiaokang Yi^1^ Shihan Jia^1^ Yasheng Li^2*^ Lei zhang^3*^ Jiabin Li^2*^ Jinxing Song^1*^

^1^ The Key Laboratory of Biotechnology for Medicinal and Edible Plant Resources of Jiangsu Province, School of Life Sciences, Jiangsu Normal University, Xuzhou 221116, China

2 Department of Infectious Diseases & Anhui Province Key Laboratory of Infectious Diseases, The First Affiliated Hospital of Anhui Medical University, Hefei, 230022, China.

3 Basic Medical Research Center，Shandong Provincial Third Hospital, Jinan, 250031 China

4. Anhui Key Laboratory of Infection and Immunity, Department of Microbiology, School of Basic Medicine, Bengbu Medical University, Bengbu, Anhui 233030, PR China.

*Correspondence: [liyasheng@ahmu.edu.cn](mailto:liyasheng@ahmu.edu.cn); [leizhang@sdu.edu.cn](mailto:leizhang@sdu.edu.cn); [lijiabin@ahmu.edu.cn](mailto:lijiabin@ahmu.edu.cn); [zdsongjinxing@jsnu.edu.cn](mailto:zdsongjinxing@jsnu.edu.cn);

^#^ These authors contributed equally to this work.

**Materials and Methods**

**Strains and Cultivation Conditions**

*A. fumigatus* A1160 was obtained from the Fungal Genetics Stock Center (FGSC) and used to generate the *ΔcrzA*, *ΔcchA*, *ΔcnaA*, and *Tet-hsp90* strains. The *Tet-hsp90* strain was cultured in medium supplemented with 1 µg/mL doxycycline. The media used in this study included YAG (2% glucose, 0.5% yeast extract, and trace elements), Minimal Medium (MM; 1% glucose, salts, and trace elements), and YUU (0.5% yeast extract, 2% dextrose, trace elements, 1.2% uracil, and 1.1% uridine). The trace element solution (100 mL) contained 2.20 g ZnSO₄·7H₂O, 1.10 g H₃BO₃, 0.50 g MnCl₂·4H₂O, 0.16 g FeSO₄·7H₂O, 0.16 g CoCl₂·5H₂O, 0.16 g CuSO₄·5H₂O, 0.11 g (NH₄)₆Mo₇O₂₄·4H₂O, and 5.00 g Na₄EDTA. Strain A1160 was routinely cultured on YUU at 37 °C. Conidia were harvested from plates after 48 h of incubation at 37 °C, diluted as required, and counted using a Neubauer hemocytometer. All media components were purchased from Shanghai Shenggong Biotechnology Co., Ltd. (Shanghai, China).

**Construction of The *Tet*-*hsp90* Strain**

The pyrithiamine resistance cassette and the Tet-On system were amplified from plasmid pCH008 using the primer pair *Tet-hsp90* SF/SR. Approximately 1 kb fragments immediately upstream and downstream of the *hsp90* start codon (ATG) were amplified using the primer pairs *Tet*-*hsp9*0 P1/P3 and *Tet*-*hsp90* P4/P6, respectively. These fragments were fused by PCR using primers *Tet*-*hsp90* P2/P5, and the resulting PCR product was used to transform strain A1160. Homologous integration was verified by PCR using the primer pairs *Tet*-*hsp90* P1/*tet*-verification up and *tet*-verification down/*Tet*-*hsp90* P6.

**Measurement of Reactive Oxygen Species**

A total of 1 × 10⁷ spores were incubated in 100 mL YAG medium supplemented with 1 µg/mL doxycycline at 37 °C for 18 h with shaking at 220 rpm. Then, 20 μM 2′,7′-dichlorodihydrofluorescein diacetate (H_2_DCFDA; Invitrogen) was added to the medium and incubated at 37°C for 1 h. After that, the mycelia were harvested and washed three times with the distilled water to remove extracellular H_2_DCFDA. The filtered mycelia were then ground in liquid nitrogen and suspended in PBS. The resulting supernatant was collected by centrifugation at 15,000 × *g* and 4°C for 10 min. Fluorescence was measured using a SpectraMax M2 reader (Molecular Devices, USA), with an excitation wavelength of 504 nm and an emission wavelength of 524 nm. The fluorescence intensity was normalized to the protein concentration of the sample, which was measured using a Bio-Rad protein assay kit.

**RNA Extraction and Quantitative Real-Time PCR (RT-qPCR)**

Spores of *A. fumigatus* were inoculated into liquid MM supplemented with 1 µg/mL doxycycline and incubated at 37 °C with shaking at 200–250 rpm for 16 h to allow spore germination. After incubation, mycelia were immediately collected and rapidly frozen in liquid nitrogen to preserve RNA integrity under RNase-free conditions. Total RNA was extracted using TRIzol reagent (Vazyme, Nanjing, China) according to the manufacturer's instructions. The extracted RNA was assessed for quality, and RNA of satisfactory quality was reverse transcribed into cDNA using a PrimeScript RT Reagent Kit (Vazyme, Nanjing, China). Quantitative real-time PCR (RT-qPCR) was then performed using SYBR Green dye (Vazyme, Nanjing, China), with ACT1 as the internal reference gene. Each reaction included at least three technical replicates. Data analysis was carried out using the 2^-ΔΔCT^ method to compare gene expression levels between the experimental and control groups, thereby assessing the impact of nootkatone on target gene expression.

**Immune Cell Cytokine Detection**

*A. fumigatus* spores were cultured on YAG agar plates supplemented with 1 µg/mL doxycycline for 5 days, harvested, filtered to remove hyphal fragments, and resuspended in PBS or sterile water. Spore concentrations were determined using a hemocytometer and adjusted to 1 × 10⁷ spores/mL. Macrophages were maintained in RPMI 1640 medium containing 10% FBS, 0.5 µg/mL doxycycline, and 1% penicillin–streptomycin at 37 °C with 5% CO₂. Cells were infected with spores for 4 h, with uninfected cells serving as controls.Total RNA was extracted using an RNA isolation kit, and cDNA was synthesized by reverse transcription. RT-qPCR was performed using SYBR Green chemistry, with β-actin as the internal control. The expression levels of IL-1β, IL-6, and TNF-α were quantified using the 2⁻^ΔΔCt^ method. Each experiment included at least three biological replicates and three technical replicates. Statistical analyses were conducted using GraphPad Prism, and significance was determined by one-way ANOVA (*p* < 0.01).

**Western blot analysis**

Macrophages were infected with WT or *Tet-hsp90* spores for 0, 2, or 4 h. Cells were then collected, washed twice with ice-cold PBS, and lysed in RIPA buffer containing protease and phosphatase inhibitors. Lysates were incubated on ice for 30 min and clarified by centrifugation at 12,000 × g for 15 min at 4 °C. Protein concentrations were determined using a BCA assay. Equal amounts of protein were separated by SDS–PAGE, transferred to PVDF membranes, and blocked with 5% nonfat milk in TBST for 1 h at room temperature. Membranes were incubated overnight at 4 °C with primary antibodies against phosphorylated and total SYK, NF-κB p65, p38 MAPK, ERK1/2, and JNK, followed by incubation with HRP-conjugated secondary antibodies for 1 h at room temperature. Protein signals were detected using enhanced chemiluminescence. Band intensities were quantified with ImageJ, and phosphorylation levels were calculated as the ratio of phosphorylated to total protein and normalized to β-actin. All experiments were performed at least three times, and representative results are shown.
